# Draft Genome Sequence of the Mucin Degrader Clostridium tertium WC0709

**DOI:** 10.1128/MRA.00642-21

**Published:** 2021-08-12

**Authors:** Eliana Musmeci, Francesco Candeliere, Alberto Amaretti, Maddalena Rossi, Stefano Raimondi

**Affiliations:** a Department of Life Sciences, University of Modena and Reggio Emilia, Modena, Italy; b BIOGEST-SITEIA, University of Modena and Reggio Emilia, Modena, Italy; University of Maryland School of Medicine

## Abstract

The draft genome sequence of Clostridium tertium WC0709, a gut bacterium able to use mucin in pure culture as the sole carbon and nitrogen source, is presented here. The genome sequence of *C. tertium* will provide valuable references for comparative genome analysis and for studying the relationship with the host.

## ANNOUNCEMENT

Clostridium tertium was first described by Henry in 1918 as Bacillus tertius (1) and was reclassified as Clostridium tertium in 1923 (2). It is a Gram-positive, anaerobic but aerotolerant, nontoxigenic (3) bacterium that forms spores only under anaerobic conditions. It can be isolated from soil, animals, and the human gastrointestinal tract (4). *C. tertium* is an uncommon human pathogen responsible for clinically significant bacteremia (5–7).

The strain *C. tertium* WC0709 was identified as a mucinolytic bacterium of the human gut in a previous study approved by the local institutional review board (reference number 125-15, Comitato Etico Provinciale, Azienda Policlinico di Modena, Italy) (8). Medium containing mucin as the sole carbon and nitrogen source (MM) was utilized in enrichment cultures inoculated with fresh feces of healthy adults. The isolated strains were taxonomically assigned by 16S rRNA gene sequencing. *C. tertium* WC0709 grew in pure culture utilizing mucin as the sole carbon and nitrogen source, without the need of any cross-feeding interaction with other intestinal bacteria that make possible the use of this complex glycoprotein.

The strain was cultivated in minimal medium (MM) at 37°C for 48 h under strictly anaerobic conditions. Biomass was collected by centrifugation, and the genomic DNA was extracted with a DNeasy blood and tissue kit (Qiagen GmbH, Düsseldorf, Germany). Before DNA purification, the pretreatment for Gram-positive bacteria was followed by incubation for 2 h at 37°C with 2-fold the volume of enzymatic lysis buffer and for 1 h at 56°C with 2-fold the volume of proteinase K and buffer AL. The DNA concentration was quantified with a Qubit 3.0 fluorimeter (Thermo Fisher Scientific, Waltham, MA, USA). The library was generated with the NEBNext Ultra II FS DNA library prep kit (New England BioLabs, Ipswich, MA, USA) and sequenced with an Illumina NovaSeq 6000 device by Eurofins Genomics (Ebersberg, Germany).

The sequencing run produced 15,916,139 paired-end reads that were 150 bp long. The raw reads were checked for quality with FastQC v0.11.8 (9). To trim Illumina adapters and remove reads with a quality score lower than 20, Cutadapt v1.16 was used with the following parameters: overlap = 15, minimum length = 30, and quality cutoff = 20 (10). The trimmed reads were verified again with FastQC and then assembled with SPAdes v3.13.0 with the parameters -careful, -cov-cutoff auto, and -k auto (11). The quality of the assembly was evaluated using QUAST v5.0.2 (12). Trimming, quality checking, and assembly were performed on the Galaxy platform (usegalaxy.eu) (13). The annotation was carried out with the NCBI Prokaryotic Genome Annotation Pipeline (PGAP) v5.2 with the methods “best-placed reference protein set” and “GeneMarkS-2+” (14). The taxonomy attribution was confirmed with SpeciesFinder (https://cge.cbs.dtu.dk/services/SpeciesFinder) (15). For all the tools, default parameters were applied unless otherwise specified.

The assembly produced a draft genome sequence encompassing 42 contigs (≥200 nucleotides [nt], 102 in total). The *N*_50_ length is 1,159,315 bp, while the *L*_50_ count is 2. The estimated genome size is 3,895,757 bp with a 27.72% G+C content and 1,230× coverage. A total of 3,578 coding sequences were annotated, comprising 10 rRNA genes (three 5S, four 16S, and three 23S) and 69 tRNAs.

### Data availability.

The sequence data were deposited in GenBank under BioProject accession number PRJNA737738 with BioSample accession number SAMN19707909 and GenBank accession number JAHLZG000000000. The raw reads have been deposited in the Sequence Read Archive (SRA) under accession number SRR14829814.
